# Perfusion and visceral organ protection in patients with thoraco-abdominal aneurysm repair – our experiences

**DOI:** 10.1186/1749-8090-8-S1-O216

**Published:** 2013-09-11

**Authors:** P Mardešić, D Zovko, M Milković, D Antunović, ZA Korda, A Škopljanac-Mačina, I Alfirević, V Juranko

**Affiliations:** 1 Magdalena – Clinic for Cardiovascular Diseases, Medical University of Osijek, Cardiosurgery Department, Cardiovascular Perfusion Unit, Krapinske Toplice, Croatia

## Background

We report our experience with 8 patients with thoraco-abdominal aneurysm repair in period of 30 months (June 2010 till December 2012), in MAGDALENA - Clinic for Cardiovascular Diseases, Medical University of Osijek.

## Methods

We conducted a retrospective observational analysis of our experience with different approaches and perfusion techniques in patients with thoraco-abdominal aneurysm.

- Group 1: 3 patients: aortic cross-clamp, with left heart bypass, without selective organ perfusion

- Group 2: 2 patients: aortic cross-clamp, without left heart bypass, selective organ perfusion

- Group 3: 1 patient: aortic cross-clamp, with left heart bypass, selective organ perfusion

- Group 4: 2 patients: aortic cross-clamp, without left heart bypass, without selective organ perfusion

## Results

Between June 2010 and December 2012, we performed 8 patients with zero mortality and one late post-operative complication, paraplegia after 48 hours. Left heart bypass duration 57 ± 14 minutes, aortic cross-clamp time duration 48 ± 13 minutes, median ICU stay was 95 hours, average hospital stay was 12 ± 3 days.

## Conclusions

Thoraco-abdominal aneurysm repair with appropriate choice of operative technique and possibility of using left heart bypass and/or selective organ protection provides good results by means of no mortality and low rate of postoperative complications and morbidity.

